# Elevated admission lactate levels in the emergency department are associated with increased 30-day mortality in non-trauma critically ill patients

**DOI:** 10.1186/s13049-020-00777-y

**Published:** 2020-08-17

**Authors:** Michael Bernhard, Stephanie Döll, Andre Kramer, Lorenz Weidhase, Thomas Hartwig, Sirak Petros, André Gries

**Affiliations:** 1 Emergency Department, University Hospital of Düsseldorf, Heinrich-Heine University, Moorenstrasse 5, 40225 Düsseldorf, Germany; 2 Emergency Department, Helios Hospital Erfurt, Erfurt, Germany; 3 Department of Anaesthesiology and Intensive Care Medicine, University Hospital Leipzig, Leipzig, Germany; 4 Medical Intensive Care Unit, University Hospital of Leipzig, Leipzig, Germany; 5 Emergency Department, University Hospital of Leipzig, Leipzig, Germany

**Keywords:** Admission lactate, Non-trauma critically ill, Resuscitation room, Emergency department, Mortality

## Abstract

**Background:**

Elevated blood lactate levels were reported as useful predictors of clinical outcome and mortality in critically ill patients. To identify higher-risk patients, this investigation evaluated the relationship between patient mortality and admission lactate levels during the management of non-trauma critically ill patients in the emergency department (ED).

**Methods:**

In this prospective, single centre observational study in a German university ED, all adult patients who were admitted to the ED resuscitation room were evaluated between September 1, 2014 and August 31, 2015. Blood samples for blood gas analysis, including lactate levels, were obtained immediately at admission. Study endpoint was 30-day mortality.

**Results:**

During the study period, 532 patients were admitted to the resuscitation room of the ED. The data of 523 patients (98.3%) were available. The overall 30-day mortality was 34.2%. Patients presenting to the resuscitation room with admission lactate levels < 2.0 mmol/l had a 30-day mortality of 22.7%, while admission lactate levels above 8.0 mmol/l were associated with higher mortality (8.0–9.9 mmol/l: OR: 2.83, 95%CI: 1.13–7.11, *p* = 0.03, and ≥ 10 mmol/l: OR: 7.56, 95%CI: 4.18–13.77, *p* < 0.001).

**Conclusion:**

High lactate levels at admission are associated with an increased 24-h and 30-day mortality. These measurements may be used not only to predict mortality, but to help identify patients at risk for becoming critically ill. The breakpoint for mortality may be an ALL ≥8.0 mmol/l.

## Introduction

Critically ill, non-trauma patients in the emergency department (ED) are a major, if under-represented group in the body of scientific literature [1]. Still, a significant amount of evidence demonstrates that initial lactate levels are useful predictor of organ failure, clinical outcome and mortality in ED patients suffering e.g. from sepsis [2], pneumonia [3], and gastrointestinal bleeding [4].

While lactate is not a direct measure of tissue perfusion, it can serve as a surrogate, as hyperlactatemia may represent different causes associated with worse outcome. Should a patient present to the ED with elevated lactate levels (> 2.0 mmol/l), it is imperative that those values be remeasured within a given time interval to monitor and guide normalization during resuscitation, as was recommended by the publication of the International Guidelines for Management of Sepsis and Septic Shock [5, 6]. The surviving sepsis campaign recommended in the 2018 update “Hour-1 bundle” that lactate levels be remeasured within 1 h of admission into the ED for all patients with suspected sepsis or septic shock [7]. The goal being to manage the sepsis as quick as possible. Further data from critically ill ED patients who were admitted to the resuscitation room showed that lactate dynamics and time weighted average lactate may predict survival beyond 30 days [8]. Additionally, blood lactate measurements are used to detect patients in critical conditions: e.g. occult shock, organ hypoperfusion or oxygen debt, in the ED.

There are still unanswered questions regarding the influence of admission lactate levels (ALL), namely whether or not ALL is a good predictor of mortality in a general cohort of critically ill non-trauma patients admitted to the resuscitation room of an ED.

The aim of this investigation was to evaluate the association between ALL and mortality among critically ill non-trauma patients presenting to the resuscitation room of a tertiary academic medical center’s ED in an effort to better identify patients at high risk of death.

## Patients and methods

We conducted a prospective, single-centre observational study from September 1, 2014 to August 31, 2015, set in the ED at Leipzig University Hospital, Leipzig, Germany. The study protocol was approved by the Ethics Committee of the Medical Faculty of the University of Leipzig, Germany (264–14-25,082,014 and amendment 478/16-EK).

### Study population

All patients ≥18 years of age who met the resuscitation room’s admission criteria (Table 1) were evaluated consecutively. Age, gender, and vital functions [systolic blood pressure, heart rate, temperature, oxygen saturation, respiratory rate, Glasgow coma scale (GCS)] were documented at resuscitation room admission. Out-of-hospital emergency medical services (EMS) interventions were documented, including if a patient was intubated, as well as whether mechanical ventilation, cardiopulmonary resuscitation or just support during the hospital admission were required. As part of our ED resuscitation room protocol, a blood gas analysis including lactate levels was performed in all patients within 15 min of hospital admission (Blood Gas analyzer: ABL800FLEX XQ, Radiometer, Germany). We used venous and arterial blood gas samples, because the results of both showed an excellent agreement concerning lactate levels [9, 10]. Depending on the level of admission lactate levels (ALL), patients were stratified into the following categories: 0.0–1.9 mmol/l (ALL I), 2.0–3.9 mmol/l (ALL II), 4.0–5.9 mmol/l (ALL III), 6.0–7.9 mmol/l (ALL IV), 8.0–9.9 mmol/l (ALL V), and ≥ 10.0 mmol/l (ALL VI). In an additional analysis, patients were divided into the two groups: those who were mechanically ventilated and those who were not. In yet another analysis, patients were divided into the two ALL categories < 4.0 mmol/l and ≥ 4.0 mmol/l according to Casserly et al. [2], and both groups were further classified into hypotensive (systolic blood pressure (SBP) ≤90 mmHg) and non-hypotensive patients (SBP > 90 mmHg) at hospital admission.

**Table 1:** Resuscitation room admission criteria^a^ [1],

Airway and breathing problems (“airway” and “breathing”)	
- airway obstruction (e.g. tongue swelling) - respiratory insufficiency with high respiratory rate (with respiratory weakness) or low oxygen saturation - necessity for invasive airway management - invasive and non-invasive mechanical ventilation	
Circulation problems (“circulation”)	
- cardiovascular insufficiency (e.g. hypotension, shock of any origin) - state after or under cardiopulmonary resuscitation - dysrhythmias - bleeding	
Unconsciousness or neurological deficit (“disability”)	
- ongoing unconsciousness of any origin	
Critical physical state (“environment”)	
- intoxication with an ABCDE problem - rhabdomyolysis - hypothermia	

^a^Additional other resuscitation room activation criteria may exist and activation depends on the attending physician in charge

### Study outcomes

The primary outcome was death from any cause within 30 days after hospital admission. The 24-h mortality for the different ALL categories was also analyzed.

### Statistical analysis

Continuous variables are presented as mean (±SD) or numbers and percentages. We used the Student’s t-test or the Mann-Whitney-U-test for numerical variables and the Chi^2^-test for categorical variables. Odds ratio (OR) and corresponding 95% confidence intervals (CI) are reported. All analyses were performed using the SAS software, version 9.3 (SAS Institute Inc., Cary, NC, USA). A two-sided *p* value of < 0.05 was considered statistically significant.

## Results

A total of 532 patients fulfilled the admission criteria of the ED resuscitation room, of whom full data sets were available for analysis in 523 patients (98.3%).

### Patient population

The mean age of patients was 68 ± 18 years and 57.9% were males. Clinical characteristics of the cohort at ED admission are shown in Tables 2 and 3. The mean systolic blood pressure was 135 ± 57 mmHg. 22% of patients were hypotensive at hospital admission and 7.5% of patients were undergoing cardiac arrest at hospital admission. The mean heart rate was 96 ± 38 beats per minute and the mean body temperature (tympanic) was 36.2 ± 1.3 °C. The mean respiratory rate was 20 ± 10 per minute and the mean arterial oxygen saturation was 92 ± 23%. Mechanical ventilation was already instituted at hospital admission in 36.5% of the patients, with 29.6% on invasive and 6.9% on non-invasive ventilation. The mean GCS was 8 ± 5. The 24-h mortality was 8.0% and the overall 30-day mortality was 34.2%.

**Table 2:** Vital functions and admission lactate levels

	Admission lactate level category (mmol/l)						
	0.0–1.9	2.0–3.9	4.0–5.9	6.0–7.9	8.0–9.9	≥10	all
no. (%)	**154 (29.4%)**	**146 (27.9%)**	**74 (14.1%)**	**43 (8.2%)**	**22 (4.2%)**	**84 (16.1%)**	**523 (100.0%)**
male no. (%)	85 (55.2%)	80 (54.8%)	45 (60.8%)	22 (51.1%)	13 (59.1%)	58 (69.4%)	303 (57.9%)
Age – yr	68.6 ± 17.6	67.8 ± 17.5	69.7 ± 15.3	67.4 ± 16.3	72.4 ± 16.7	63.9 ± 16.4	67.8 ± 17.6
SBP - mmHg	145.5 ± 45.9	136.5 ± 44.9	130.7 ± 44.7	133.4 ± 57.1	121.2 ± 64.5	110.3 ± 66.0	134.7 ± 56.6
Hypotensive no. (%)	11 (7.1%)	21 (14.4%)	14 (18.9%)	12 (27.9%)	10 (45.5%)	47 (56.0%)	115 (22.0%)
CPR adm.no. (%)	2 (1.3%)	5 (3.4)	1 (1.4%)	2 (4.7%)	3 (13.6%)	27 (32.1%)	39 (7.5%)
HR (X/min)	92.8 ± 28.2	94.9 ± 32.6	96.1 ± 35.7	108.6 ± 32.9	106.9 ± 48.0	96.0 ± 52.0	96.0 ± 38.0
Temp tymp. - °C	36.5 ± 14.5	36.2 ± 13.1	36.4 ± 15.4	36.4 ± 11.8	36.1 ± 15.5	35.4 ± 15.5	36.2 ± 14.3
Oxygen sat. - %	93.1 ± 13.6	91.6 ± 14.6	92.4 ± 9.3	87.6 ± 23.4	93.1 ± 28.6	88.7 ± 39.1	91.5 ± 22.6
RR - (x/min)	19.1 ± 9.5	20.3 ± 10.3	21.9 ± 11.5	23.7 ± 10.3	19.6 ± 9.2	16.4 ± 7.5	19.8 ± 10.0
GCS	9.8 ± 4.8	9.2 ± 5.0	9.2 ± 5.1	8.5 ± 4.9	5.7 ± 4.3	4.2 ± 2.9	8.4 ± 5.1
Shock Index	0.7 ± 0.3	0.8 ± 0.4	0.8 ± 0.5	0.9 ± 0.5	1.1 ± 0.6	1.0 ± 0.7	0.8 ± 0.5
Adm. MV no. (%)	43 (27.9%)	39 (26.7%)	26 (35.1%)	9 (20.9%)	12 (54.5%)	62 (73.8%)	191 (36.5%)
Invasive no. (%)	27 (17.5%)	29 (19.9%)	18 (24.3%)	7 (16.3%)	12 (54.5%)	62 (73.8%)	155 (29.6%)
Non-invasive no. (%)	16 (10.4%)	10 (6.8%)	8 (10.8%)	2 (4.6%)	0 (0.0%)	0 (0.0%)	36 (6.9%)
24 h mortality no. (%)	3 (1.9%)	2 (1.4%)	3 (4.1%)	4 (9.3%)	3 (13.6%)	27 (31.8%)	42 (8.0%)
30 d mortality no. (%)	35 (22.7%)	39 (26.7%)	21 (28.4%)	16 (37.2%)	10 (45.5%)	58 (69.0%)	179 (34.2%)

Values are mean with standard deviation; number (no.) and percentage (%), *yr* years, *SBP* systolic blood pressure, CPR adm. ongoing cardiopulmonary resuscitation at hospital admission, *HF* heart rate, *Temp tymp.* temperature tympanic, *Sat.* arterial oxygen saturation, *RR* respiratory rate (spontaneous and mechanical ventilation), *GCS* Glasgow Coma Scale, *Adm. MV* mechanical ventilation at hospital admission, invasive = endotracheal intubation, supraglottic airway device, tracheostomy, *d* days, *h* hour

**Table 3:** Patient characteristics and admission lactate levels

	Admission lactate level category (mmol/l)						
	0.0–1.9	2.0–3.9	4.0–5.9	6.0–7.9	8.0–9.9	≥10	all
no. (%)	**154 (29.4%)**	**146 (27.9%)**	**74 (14.1%)**	**43 (8.2%)**	**22 (4.2%)**	**84 (16.1%)**	**523 (100.0%)**
Sepsis	**15 (9.7%)**	**10 (6.8%)**	**12 (16.2%)**	**6 (14.0%)**	**1 (4.5%)**	**10 (11.9%)**	**54 (10.3%)**
Lower respiratory tract	9 (5.8%)	5 (3.4%)	4 (5.4%)	4 (9.3%)	0 (0.0%)	5 (6.0%)	27 (5.2%)
Urinary tract infection	5 (3.2%)	3 (2.1%)	2 (2.7%)	2 (4.7%)	1 (4.5%)	0 (0.0%)	13 (2.5%)
Intra-abdominal	0 (0.0%)	0 (0.0%)	2 (2.7%)	0 (0.0%)	0 (0.0%)	1 (1.2%)	3 (0.6%)
Other	1 (0.6%)	2 (1.4%)	4 (5.4%)	0 (0.0%)	0 (0.0%)	4 (4.8%)	11 (2.1%)
Lung disease	**36 (23.4%)**	**23 (15.8%)**	**11 (15.1%)**	**7 (16.3%)**	**3 (13.6%)**	**6 (7.1%)**	**86 (16.4%)**
COPD	15 (9.7%)	13 (8.9%)	4 (5.5%)	3 (7.0%)	2 (9.1%)	4 (4.8%)	41 (7.8%)
Pneumonia	14 (9.1%)	0 (0.0%)	5 (6.8%)	4 (9.3%)	0 (0.0%)	1 (1.2%)	24 (4.6%)
Pneumothorax	1 (0.6%)	1 (0.7%)	0 (0.0%)	0 (0.0%)	0 (0.0%)	0 (0.0%)	2 (0.4%)
Bolus	1 (0.6%)	1 (0.7%)	1 (1.4%)	0 (0.0%)	0 (0.0%)	1 (1.2%)	4 (0.8%)
Aspiration	1 (0.6%)	5 (3.4%)	1 (1.4%)	0 (0.0%)	1 (4.5%)	0 (0.0%)	8 (1.5%)
Airway bleeding	4 (2.6%)	3 (2.1%)	0 (0.0%)	0 (0.0%)	0 (0.0%)	0 (0.0%)	7 (1.3%)
Neurological disease	**63 (40.9%)**	**45 (30.8%)**	**20 (27.1%)**	**13 (30.2%)**	**6 (27.3%)**	**15 (17.9%)**	**162 (31.0%)**
Stroke	19 (12.3%)	10 (6.8%)	3 (4.1%)	0 (0.0%)	1 (4.5%)	0 (0.0%)	33 (6.3%)
Seizure	1 (0.6%)	4 (2.7%)	8 (10.8%)	8 (18.6)	4 (18.2%)	9 (10.7%)	34 (6.5%)
Intracerebral bleeding	18 (11.7%)	11 (7.5%)	4 (5.4%)	4 (9.3%)	0 (0.0%)	2 (2.4%)	39 (7.5%)
Intoxication	20 (13.0%)	14 (9.6%)	2 (2.7%)	0 (0.0%)	1 (4.5%)	0 (0.0%)	37 (7.1%)
Unconsciousness^a^	5 (3.2%)	4 (2.7%)	2 (2.7%)	1 (2.3%)	0 (0.0%)	4 (4.8%)	16 (3.1%)
Psychiatric disorder	0 (0.0%)	2 (1.4%)	1 (1.4%)	0 (0.0%)	0 (0.0%)	0 (0.0%)	3 (0.6%)
Cardiovascular disease	**32 (20.8%)**	**52 (35.6%)**	**26 (35.3%)**	**10 (23.2%)**	**10 (45.4%)**	**42 (50.0%)**	**172 (32.9%)**
Pulmonary edema	8 (5.2%)	10 (6.8%)	5 (6.8%)	1 (2.3%)	1 (4.5%)	0 (0.0%)	25 (4.8%)
Congestive heart failure	9 (5.8%)	15 (10.3%)	6 (8.1%)	4 (9.3%)	2 (9.1%)	6 (7.1%)	42 (8.0%)
arhythmia	0 (0.0%)	7 (4.8%)	2 (2.7%)	0 (0.0%)	1 (4.5%)	1 (1.2%)	11 (2.1%)
Myocardial infarction	6 (3.9%)	13 (8.9%)	7 (9.5%)	4 (9.3%)	3 (13.6%)	13 (15.5%)	46 (8.8%)
Pulmonary embolism	4 (2.6%)	6 (4.1%)	1 (1.4%)	0 (0.0%)	1 (4.5%)	12 (14.3%)	24 (4.6%)
Aortic dissection	3 (1.9%)	1 (0.7%)	3 (4.1%)	0 (0.0%)	0 (0.0%)	0 (0.0%)	7 (1.3%)
Cardiac arrest, unknown reason	2 (1.3%)	0 (0.0%)	2 (2.7%)	1 (2.3%)	2 (9.1%)	10 (11.9%)	17 (3.3%)
Gastrointestinal disease	**3 (1.9%)**	**8 (5.5%)**	**1 (1.4%)**	**3 (7.0%)**	**0 (0.0%)**	**6 (7.1%)**	**21 (4.0%)**
Lower GIB	0 (0.0%)	1 (0.7%)	0 (0.0%)	0 (0.0%)	0 (0.0%)	0 (0.0%)	1 (0.2%)
Upper GIB	2 (1.3%)	5 (3.4%)	1 (1.4%)	3 (7.0%)	0 (0.0%)	5 (6.0%)	16 (3.1%)
RAAA	1 (0.6%)	2 (1.4%)	0 (0.0%)	0 (0.0%)	0 (0.0%)	1 (1.2%)	4 (0.8%)
Others	**5 (3.2%)**	**8 (5.5%)**	**4 (5.5%)**	**4 (9.3%)**	**2 (9.1%)**	**5 (6.0%)**	**28 (5.4%)**
Drowning	1 (0.6%)	0 (0.0%)	0 (0.0%)	1 (2.3%)	0 (0.0%)	1 (1.2%)	3 (0.6%)
Hyperthermia	0 (0.0%)	1 (0.7%)	0 (0.0%)	0 (0.0%)	0 (0.0%)	0 (0.0%)	1 (0.2%)
Hypothermia	0 (0.0%)	0 (0.0%)	1 (1.4%)	0 (0.0%)	0 (0.0%)	1 (1.2%)	2 (0.4%)
Exsiccosis	0 (0.0%)	1 (0.7%)	0 (0.0%)	0 (0.0%)	1 (4.5%)	0 (0.0%)	2 (0.4%)
Renal failure	1 (0.6%)	2 (1.4%)	2 (2.7%)	0 (0.0%)	1 (4.5%)	1 (1.2%)	7 (1.3%)
Other	3 (1.9%)	4 (2.7%)	1 (1.4%)	3 (7.0%)	0 (0.0%)	2 (1.4%)	13 (2.5%)

Values as number (no.) and percentage (%), ^a^unknown origin, *GIB* gastrointestinal bleeding, *RAAA* ruptured abdominal aortic aneurysm

### Admission lactate levels

Patient characteristics stratified according to categories of the ALL were presented in Tables 2 and 3.

**Fig. 1:**
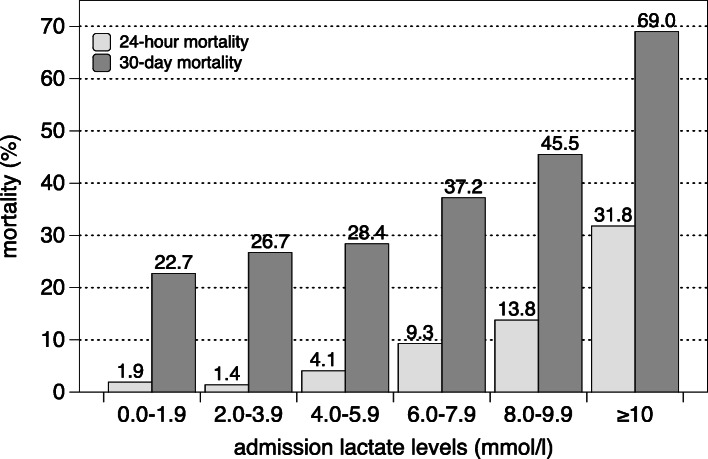
24-h mortality and 30-day mortality compared to admission lactate level category

**Fig. 2:**
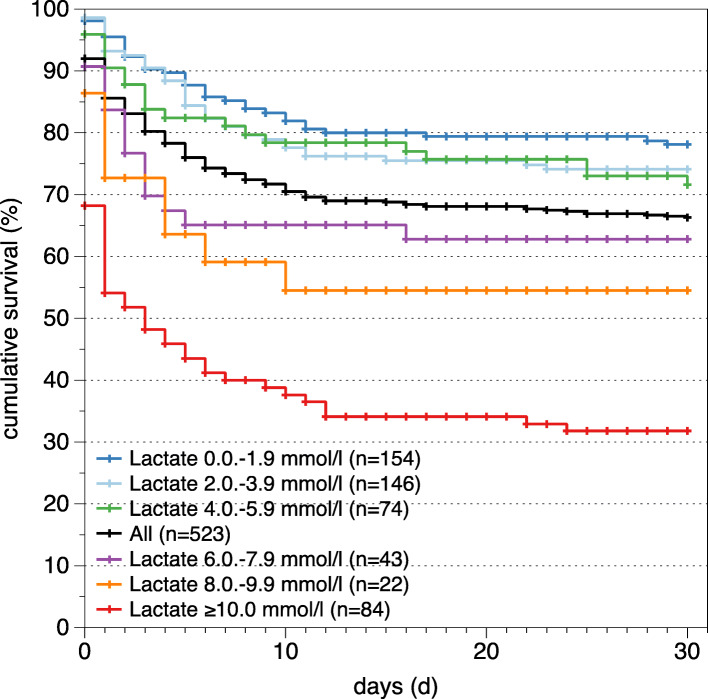
Kaplan-Meier survival curve for admission lactate level categories

The 24-h mortality and 30-day mortality increased with higher ALL (Fig. 1). In comparison to patients with a normal ALL (ALL I: 0.0–1.9 mmol/l), patients in ALL categories IV (8.0–9.9 mmol/l) and V (≥10.0 mmol/l) had a significantly higher mortality (OR: 2.83, 95% confidence interval (95%CI): 1.13–7.11, *p* = 0.03 vs. OR: 7.56, 95%CI: 4.18–13.77, *p* < 0.001) (Table 4). Patients of ALL categories II-IV did not show a higher mortality in comparison to patients with normal ALL (ALL II: 2.0–3.9 mmol/l, OR: 1.24, 95%CI: 0.73–2.10 vs. ALL III: 4.0–5.9 mmol/l, OR: 1.35, 95%CI: 0.72–2.53 vs. ALL IV: 6.0–7.9 mmol/l, OR: 2.02, 95%CI: 0.98–4.16). The Kaplan-Meier survival curve for patients in every ALL category is presented in Fig. 2.

**Table 4:** Admission lactate levels and mortality

	Admission lactate level category (mmol/l)						
	0.0–1.9	2.0–3.9	4.0–5.9	6.0–7.9	8.0–9.9	≥10	***P***
no. (%)	**154 (29.4%)**	**146 (27.9%)**	**74 (14.1%)**	**43 (8.2%)**	**22 (4.2%)**	**84 (16.1%)**	
OR	1 (Reference)	1.24	1.35	2.02	2.83^a^	7.56^b^	^a^*p* = 0,03; ^b^*p* < 0.001 vs. reference
95%CI	–	0.73–2.10	0.72–2.53	0.98–4.16	1.13–7.11	4.18–13.77	
30-day mortality [n (%)]	35 (22.7%)	39 (26.7%)	21 (28.4%)	16 (37.2%)	10 (45.5%)	58 (69.0%)	

*OR* odds ratio, *95% CI* 95% confidence interval

Patients with hypotension at admission had a significantly higher ALL than non-hypotensive patients (9.7 ± 6.9 vs. 4.2 ± 5.3 mmol/l, *p* = 0.0001). Non-survivors had significantly higher ALL than survivors (7.9 ± 6.6 vs. 4.1 ± 5.3 mmol/l, p = 0.0001).

Across all ALL categories, mechanically ventilated patients had a higher 30-day mortality than those who were not mechanically ventilated (Table 5).

**Table 5:** Vital functions and admission lactate levels for not ventilated and ventilated patients

	Admission lactate level category (mmol/l)						
	0.0–1.9	2.0–3.9	4.0–5.9	6.0–7.9	8.0–9.9	≥10	all
no. (%)	**154 (29.4%)**	**146 (27.9%)**	**74 (14.1%)**	**43 (8.2%)**	**22 (4.2%)**	**84 (16.1%)**	**523 (100.0%)**
Adm. No MV	125 (81.2%)	116 (79.5%)	56 (75.7%)	36 (83.7%)	10 (45.5%)	22 (26.2%)	365 (69.8%)
SBP - mmHg	148.2 ± 42.0	138.9 ± 37.7	130.6 ± 41.1	138.0 ± 48.5	119.7 ± 44.5	100.9 ± 40.5	138.4 ± 42.4
GCS	10.9 ± 4.3	10.7 ± 4.4	11.1 ± 4.4	9.6 ± 4.7	8.9 ± 4.8	7.4 ± 4.4	10.5 ± 4.5
30 d mortality no. (%)	30 (24.0%)	31 (26.7%)	16 (28.6%)	8 (22.2%)	1 (10.0%)	5 (22.7%)	91 (24.9%)
Adm, MV	29 (18.8%)	30 (20.5)	18 (24.3%)	7 (16.3%)	12 (54.5%)	62 (73.8%)	158 (30.2%)
SBP - mmHg	134.4 ± 38.4	128.2 ± 39.0	130.8 ± 47.3	107.0 ± 31.2	122.7 ± 54.1	115.6 ± 52.8	96.0 ± 38.0
GCS	4.3 ± 3.1	3.3 ± 1.6	3.3 ± 1.2	3.0 ± 0.0	3.0 ± 0.0	3.0 ± 0.3	3.3 ± 1.6
30 d mortality no. (%)	13 (44.8%)	8 (26.7%)	7 (38.9%)	3 (42.9%)	5 (41.7%)	33 (53.2%)	69 (43.7%)

Values are mean with standard deviation; number (no.) and percentage (%), *SBP* systolic blood pressure, *GCS* Glasgow Coma Scale, *Adm. MV* mechanical ventilation at hospital admission, *Adm. No MV* no mechanical ventilation at hospital admission, *d* days

**Fig. 3:**
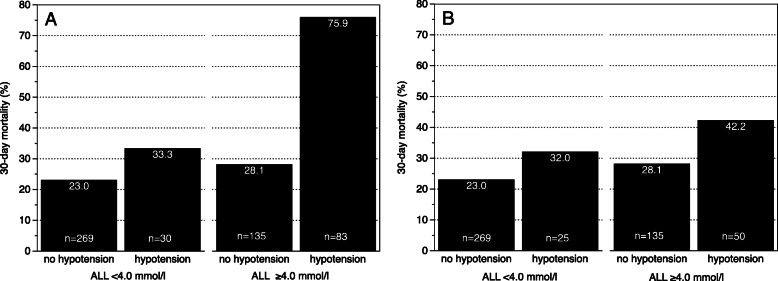
30-day mortality in patients with admission lactate levels (ALL) < 4.0 and ≥ 4.0 mmol/l divided into non-hypotensive and hypotensive subgroups: **a**) including patients under ongoing cardiopulmonary resuscitation at hospital admission, **b**) without patients under ongoing cardiopulmonary resuscitation at hospital admission

The 30-day mortality in the ALL category < 4.0 mmol/l did not differ between patients without and with hypotension (Fig. 3a) (23.0 vs. 33.3%, *p* = 0,211, two-sided Chi^2^-test). In contrast, the 30-day mortality was significantly different between patients without vs. with hypotension in the subgroup of patients with ALL category ≥4.0 mmol/l, (28.1 vs. 75.9%, *p* < 0.001).

Excluding patients who underwent cardiopulmonary resuscitation at admission (Fig. 3b), 30-day mortality in the subgroup with ALL < 4.0 mmol/l did not differ between patients without vs. with hypotension (23.0 vs. 32.0%, *p* = 0,315). In the subgroup with ALL ≥4.0 mmol/l, we found only a trend towards a higher 30-day mortality in patients with hypotension (28.1 vs. 42.2%, *p* = 0.07).

## Discussion

This prospective, single-centre, observational study demonstrated that increased levels of ALL at ED resuscitation room admission indicate a higher mortality in critically ill non-trauma patients. Patients with hypotension or cardiac arrest at hospital admission showed the highest levels. Taken together, increased ALL is strongly associated with mortality in critically ill resuscitation room patients.

Elevated lactate has been observed in a broad spectrum of patients in the ED, especially in patients suffering from infection (23%), seizure (20%), and cardiovascular disease (11%) [11]. However, while knowing the blood lactate levels was very informative in identifying critically ill patients who presented with an infection, those lactate levels did a poor job of identifying critically ill patients among those who were suffering from a seizure [11]. The results of our investigation are in line with those from Bou Chel et al. [12], finding a stepwise increase of mortality for low, intermediate, and high lactate groups in a diverse patient population. Blood lactate measurements are useful for risk assessment in patients admitted acutely to the hospital [13].

Lactate measurement plays a crucial role in early sepsis management, since lactate ≥4 mmol/l is considered a marker of sepsis severity. The surviving sepsis campaign recommends the measurement of lactate within 1 h after ED presentation for all patients with suspected sepsis or septic shock [7]. Only 10% of our cohort were suffering from sepsis. Regardless of sepsis, ALL ≥8.0 mmol/l was associated with significantly higher all-cause mortality. The breakpoint for mortality prediction seems to be an ALL ≥8.0 mmol/l. In line with the literature, a blood lactate above 10.0 mmol/l was associated with the highest mortality (OR 7.56) [14]. The lack of significance for lower lactate categories may also be due to the size of our cohort.

The strength of our study is that blood lactate was measured immediately after hospital admission during the resuscitation room course. In this regard, the recommendation of the surviving sepsis campaign was fulfilled [7], which is important because delayed resuscitation in patients with elevated lactate is associated with significantly increased risk of death [15, 16].

Blood lactate measurements are useful for risk assessment and for predicting in-hospital mortality in a diverse population [13]. In line with the retrospective study by Haas et al. [14] in a large cohort of un-selected ICU patients, severe hyperlactatemia was associated with extremely high mortality. These authors found a high mortality in patients suffering from a broad spectrum of medical disease (e.g. mesenteric ischemia, liver failure, cardiogenic shock, sepsis, cardiopulmonary resuscitation) and hyperlactatemia, especially when there was no marked lactate clearance within 12 h [14]. The breakpoint of mortality in this retrospective study was a hyperlactatemia ≥10 mmol/l.

One target of the recommendations of the surviving sepsis campaign is to normalize lactate in patients with elevated lactate. Due to the empirical nature of the presented investigation, we did not evaluate the lactate clearance and its effect on outcome. However, lactate clearance may be associated with improved outcome in heterogeneous ICU and ED patients [8, 17, 18].

Our study has several clinical implications. Firstly, elevated ALL is a surrogate parameter of adverse clinical outcome, and this may be helpful for risk stratification in critically ill patients admitted to the resuscitation room. Thereby, ALL may be a rapidly available, reliable and inexpensive tool to identify at-risk patients in the early period of ED management. Secondly, our findings supported the existing evidence that elevated ALL is associated with higher mortality in critically ill patients. Our results determine that elevated ALL should be taken into account not only when treating septic patients, but when treating all other critically ill patients. To clarify, ALL is not a “stand alone”, “one-fits all” or “the one and only” parameter, and successful treatment is dependent upon a variety of clinical and preclinical parameters (‘decision bundle’). However, ALL may be a valuable first step in the diagnostic process. It is worth mentioning that elevated ALL are also observed in patients without hypotension or cardiac arrest (e.g. occult shock). It should be kept in mind, that in line with the literature, patients with normal ALL may be critically ill [19]. Other factors aside from existing deterioration of microcirculatory (e.g. persisting cellular hypoperfusion) may have contributed to elevated lactate levels in ED patients, such as liver failure, temporary high adrenaline levels, or alcohol intake [13].

### Limitations

This study has a single-centre design with all its well-known limitations. However, despite the high number of included patients, the patient cohort showed a broad spectrum of patient diagnoses. However, our study presented a general un-selected, non-trauma cohort of critically ill patients, which represented the real-life situation in the ED. The fact that significant results were reached only for ALL ≥8.0 mmol/l in the present investigation should not lead to the interpretation that a lower ALL is not associated with mortality. If more patients were included, a finer resolution of the association between the lactate and mortality could have been possible. However that was limited by the format of the study and the unbiased way in which the patients were recruited. Despite of the large number of included patients, the heterogeneity of the diagnoses is a possible source of bias. Nevertheless, our cohort represented the real-life situation in the ED.

## Conclusion

High lactate levels at admission are associated with an increased 24-h and 30-day mortality. These measurements may be used not only to predict mortality, but to help identify patients at risk for becoming critically ill. The breakpoint for mortality may be an ALL ≥8.0 mmol/l.

## Data Availability

The data sets generated and analyzed during this study are not publicly available due to private reason but are available from the corresponding author on reasonable request.
